# The Effects of Two *Lactobacillus plantarum* Strains on Rat Lipid Metabolism Receiving a High Fat Diet

**DOI:** 10.1155/2013/135142

**Published:** 2013-12-29

**Authors:** Rastislav Salaj, Jana Štofilová, Alena Šoltesová, Zdenka Hertelyová, Emília Hijová, Izabela Bertková, Ladislav Strojný, Peter Kružliak, Alojz Bomba

**Affiliations:** ^1^Institute of Experimental Medicine, Faculty of Medicine, P. J. Šafárik University, Trieda SNP 1, 040 01 Košice, Slovakia; ^2^Department of Medical and Clinical Biochemistry, Faculty of Medicine, P. J. Šafárik University, Trieda SNP 1, 040 01 Košice, Slovakia; ^3^Department of Cardiovascular Diseases, International Clinical Research Center, St. Anne's Faculty Hospital, Masaryk University, Pekařská 53, 656 91 Brno, Czech Republic

## Abstract

The aim of our study was to evaluate the effects of the different probiotic strains, *Lactobacillus plantarum* LS/07 and *Lactobacillus plantarum* Biocenol LP96, on lipid metabolism and body weight in rats fed a high fat diet. Compared with the high fat diet group, the results showed that *Lactobacillus plantarum* LS/07 reduced serum cholesterol and LDL cholesterol, but *Lactobacillus plantarum* Biocenol LP96 decreased triglycerides and VLDL, while there was no change in the serum HDL level and liver lipids. Both probiotic strains lowered total bile acids in serum. Our strains have no significant change in body weight, gain weight, and body fat. These findings indicate that the effect of lactobacilli on lipid metabolism may differ among strains and that the *Lactobacillus plantarum* LS/07 and *Lactobacillus plantarum* Biocenol LP96 can be used to improve lipid profile and can contribute to a healthier bowel microbial balance.

## 1. Introduction

Cardiovascular disease due to atherosclerosis of the arterial vessel wall and to thrombosis is the foremost cause of premature mortality and of disability-adjusted life years in Europe and is also increasingly common in the developing countries [1]. Hyperlipidaemia is a dominant risk factor for cardiovascular diseases and the leading cause of death in many countries. Elevated serum cholesterol is generally a risk factor correlated with the development of coronary artery diseases. Dietary fat is one of the most important environmental factors associated with the incidence of those diseases; diets high in cholesterol and saturated fat have been shown to promote atherosclerosis [2]. Atherosclerosis is considered to be a modified form of chronic inflammation induced by lipids and many have followed in this path including evidence that numerous cell adhesion molecules and growth factors were determined in the atherosclerotic plaques [3]. The current drug therapy has the disadvantage owing to its undesirable side effects and cost, so there is an increasing interest in alternative approaches to lower cholesterol [4]. Diet intervention supplements have now been extensively studied to reduce risk factor for cardiovascular disease. Numerous animal experiments and human studies have reported that probiotic microorganisms display hypolipidemic effects by inhibiting cholesterol biosynthesis and decreasing low density lipoproteins [5, 6]. Food and Agriculture Organization and World Health Organization defined probiotics as “Living microorganisms which when administered in adequate amounts confer a health benefit on the host” [7]. There is currently adopted definition by FAO/WHO, but in the present research publications there are also new definitions of probiotics as follows: probiotics are living microorganisms which modulate the specific function of organism by activation of specific molecular pathways [8].

The gastrointestinal tract is one of the largest interfaces between the outside world and the human or animal internal environment. It is a diverse microenvironment with more than 500 species of bacteria. The gastrointestinal microflora maintains a microbial barrier against the development of pathogenic bacteria in the digestive tract [9]. Application of probiotics can beneficially affect the colon microflora. A large proportion of the faecal mass consists of bacteria—around 60% of faecal material. The stomach and small intestine contain only a few species of bacteria adhering to the epithelium and some other bacteria in transit [10]. Adhesion of the intestine is crucial for providing the beneficial effects of probiotics, since it may influence interaction with the host [11]. The serum lipid level is influenced by food. This fact has been known for a long time. The first record of the reduction of serum cholesterol by probiotic microorganism (milk fermented with *Lactobacillus *strain) was described by Shaper et al. [12] and Mann and Spoerry [13]. In addition, the mechanisms of the hypolipidemic activity of probiotic bacteria have been proposed to involve deconjugate bile acid through bile salt hydrolase catalysis, take up and assimilate cholesterol for stabilization of their cell membrane and binding cholesterol to cell walls of probiotics in intestine, conversion of cholesterol into coprostanol, and inhibit hepatic cholesterol and triglyceride synthesis by short chain fatty acids such as propionate and redistribution of cholesterol from plasma to the liver [6]. Bacterial species that are currently of commercial interest as probiotics mainly belong to the genera *Lactobacillus* and *Bifidobacterium* [14]. The mechanism of action of probiotics is largely unknown.

## 2. Material and Methods

### 2.1. Animals

We used male (*n* = 20) and female (*n* = 20) *Sprague Dawley* albino rats (Central Vivarium, Medical Faculty, P. J. Šafárik University, Košice, Slovakia), three months old with mean body weight of 357.25 ± 9.12 g. The rats were placed in plastic cages with tops and exposed to a 12 h light/dark cycle and maintained at a constant temperature of 22 ± 2°C and humidity of 55 ± 5%. The experiment was performed complying with ethical requirements for animal handling pursuant to Acts numbers 289/2003 and 489/2003 of Slovakia on the Care and Use of Laboratory. The experiment was approved by the Ethical Commission. The healthy experimental rats were randomly divided into 4 groups of 10 animals each (5 male and 5 female): C: control group was fed conventional laboratory diet (CLD), group 2 HFD: rats were fed high fat diet (CLD supplemented with 20% lard), group 3 LPH: rats were fed high fat diet supplemented with probiotic *Lactobacillus plantarum* LS/07, and group 4 LPP: rats were fed high fat diet supplemented with probiotic *Lactobacillus plantarum* Biocenol LP96. The rats were fed conventional laboratory diet produced by Miško Peter (Snina, Slovakia). A high fat diet was prepared from conventional laboratory diet which was supplemented with 20% lard (w/w). Drinking water was provided *ad libitum*. Food and water intake was monitored daily and body weights were recorded weekly.

### 2.2. Probiotic Strains

The probiotic strain of *Lactobacillus plantarum* LS/07 was isolated from rectal human swabs (according to Strojný et al. [15]). The probiotic strain of *Lactobacillus plantarum* Biocenol LP96 was isolated from the gut contents of healthy suckling piglets. Nemcová et al. [16, 17] published characteristic properties of *Lactobacillus plantarum* Biocenol LP96. The strains were cultured in MRS broth (Merck, Germany). The probiotic strains were provided in dose 3 × 10^9^ CFU of strain/1 mL MRS medium, prepared as night cultures at 37°C aerobically. Then 0.5 mL of lactobacilli strains was used and mixed with 9 mL of pasteurised milk. The milk (temperature was 20–22°C) was filled into screw caped bottles and was administered every day. Each rat received approximately 1.5 × 10^9^ CFU lactobacilli via the oral route.

### 2.3. Preparation of Caecal Samples

The fresh samples of caecum were taken in all experimental rats after death. Caecal samples of rats (1 g) were placed in sterile polyethylene Stomacher Lab Blender bags with sterile diluents (9 mL) of Ringer's solution and mixed in a Stomacher 400 Bag mixer (France). The series of 10-fold dilutions (from 10^−2^ to 10^−8^) were made in the same sterile diluents. The dilutions (100 *μ*L of each) were spread-plated onto agar for lactic acid bacteria MRS agar (Merck, Germany). MRS medium is selective for lactobacilli but some growth of leuconostocs and pediococci may occur. The plates for lactic acid bacteria were placed in box (Gas Pak, USA) and incubated at 37°C for 48 h. The colonies were counted and bacteria were Gram-stained in a light microscope. The numbers of colony forming units (CFU) are expressed as log_10_ CFU per gram.

### 2.4. Measurement of Faecal *β*-Glucuronidase Activity

The activity of *β*-glucuronidase was tested in faecal samples of rats as previously described method of Juskiewicz et al. [18].

### 2.5. Laboratory Analysis

Rats were killed after 10 weeks of the experiment. Animals were anaesthetised *i.m*. by Zoletil 50 mg/kg b.w. The blood samples from each rat were taken from heart by puncture. The serum was separated from the blood by centrifugation at 2500 ×g for 10 min and kept frozen at −80°C until further analysis. The blood serum was used for determination of total bile acids concentration with a commercial kit (Trinity Biotech, Ireland) and lipid parameters. Serum total cholesterol (TC), high density lipoproteins cholesterol (HDL-CH), and triglycerides (TG) were measured by using an automatic biochemical analytical system. Low density lipoprotein (LDL-CH) was calculated by Friedewald formula [19]: low density lipoprotein cholesterol = total  cholesterol − HDL  cholesterol − (TG/2.2), with all concentrations as millimoles per liter; very low density lipoprotein cholesterol = TG/2.2, with this quotient used as an estimate of VLDL cholesterol. Formula for non-HDL cholesterol = total  cholesterol − HDL cholesterol.

### 2.6. Liver and Fecal Cholesterol and Triglycerides

After an animal had been killed, the liver was removed, rinsed with physiological saline solution, blotted dry with filter paper, and weighed. Liver lipids were extracted according to the Folch et al. [20] method. Liver tissue 0.5 g was ground in 10 mL of Folch solution (chloroform : methanol = 2 : 1) for 24 hours. The homogenate was then filtered with Whatman number 2 filter paper. The organic layer was then evaporated under a nitrogen stream. The dried lipid layer was dissolved with 1 mL DMSO and then used to determine the TC and TG levels by using commercial kits (Biovendor, Czech Republic). Fecal TC and TG content were determined according to the method of Wang et al. [21].

### 2.7. Body Composition Analysis

A quantitative nuclear magnetic resonance analyser EchoMRI-700 (Medical Systems, Houston, TX) was used to measure total fat mass and lean at the end of experiments.

### 2.8. Calculated Parameters

Body weight change (BWCH) percentage for the rats was calculated as follows:

(1) $$\begin{array}{c} \text{BWCH}=\left[\frac{\left(\text{weight}\;\text{per}\;\text{week}-\text{initial}\;\text{value}\right)}{\text{initial}\;\text{value}}\right]\times 100. \end{array}$$

Feed efficiency ratio (FER) was calculated as

(2) $$\begin{array}{c} \text{FER}=\frac{\text{gain}\;\text{body}\;\text{weight}}{\text{feed}\;\text{intake}}. \end{array}$$

### 2.9. Statistical Analysis

Results are expressed as mean ± standard error of the mean (SEM). Significant differences among groups were determined using Tukey test (MINITAB for Windows 11.21). Values of *P* < 0.05 were considered significant. The relationships between the total lactobacilli counts and serum total bile acids and cholesterol were determined by linear correlation analysis.

## 3. Results

**Figure 1:**
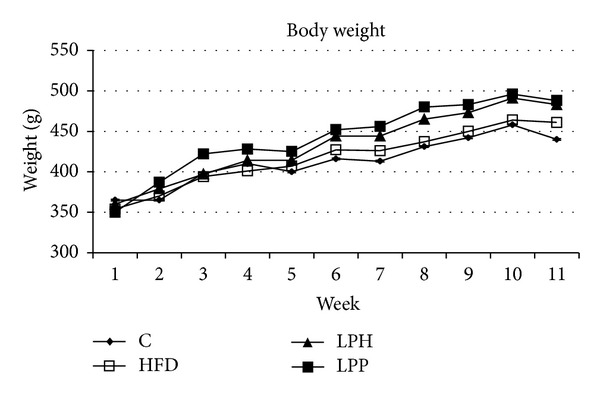
Trends of body weight. Values represent mean (*n* = 10). C: control group; HFD: high fat diet group; LPH: HFD + *Lactobacillus plantarum* LS/07; LPP: HFD + *Lactobacillus plantarum* Biocenol LP96.

The basal body weight of rats was comparable before the start of the study. The weight of all groups was increased every week, especially LPH and LPP groups (Figure 1). All rats fed the high fat diet exhibited slightly higher body weight, weight gain, and body fat, but ratio weight gain/final body weight was significantly elevated (*P* < 0.05) compared to the control rats (Table 1). The weight gain/final body weight ratio is a better parameter than the body weight and weight gain, because it minimizes the differences between male and female rats. There were no significant differences in liver weight (*P* < 0.05) among the four groups. The LPH group showed a lower liver/final body weight ratio (*P* < 0.05) than the HFD group.

The daily food intake of groups fed a high fat diet was lower (*P* < 0.05) than that of the control group but did not differ between the HFD and HFD probiotic groups. At the end of the experiment, the body weight change percentage and feed efficiency ratio were higher (*P* < 0.05) in the LPH and LPP groups than control group (Table 2).

The serum cholesterol, triglyceride, HDL-CH, and bile acid levels and calculated parameters of LDL-CH, VLDL, and non-HDL cholesterol in each group are summarized in Table 3. Serum cholesterol and LDL-CH levels were higher in the rats on high fat diet (13% and 35%, resp.) than in the control rats. Compared with the HFD group, the LPH and LPP groups had more or less decreased TC, LDL-CH, bile acids, and non-HDL levels. HDL-CH levels were not different. Hepatic and fecal lipid content was higher in high fat diet fed rats than in the control rats.

The counts of lactic acid bacteria (LAB) and activity of *β*-glucuronidase are shown in Table 4. The caecal counts of LAB were lower (*P* < 0.05) in the HFD group than in the control group. However, after treatment with *Lactobacillus plantarum *LS/07, the counts were significantly increased (*P* < 0.05) in the LPH group compared with counts in HFD group. In contrast, oral supplementation with *Lactobacillus plantarum *Biocenol LP96 increased LAB counts in the LPP group slightly compared with HFD group. After lactobacilli treatment, the activity of *β*-glucuronidase was nonsignificantly increased (*P* < 0.05) in LPH and LPP groups in comparison with HFD group.

## 4. Discussion

Epidemiological data indicate that nutrition has a major impact on human health. The intake of high amounts of fat is a major risk factor in the etiology of cardiovascular disease. Dietary lipids influence the gastrointestinal microbiota and specifically the population of lactic acid bacteria [22]. It was demonstrated that composition of gut flora may affect host's ability to harvest energy from the diet [23, 24] and this composition may produce difference in energy intake, utilization, and storage. Cani et al. [25] demonstrated that high fat feeding modulates gut microbiota and the plasma concentration of lipopolysaccharide, that is, metabolic endotoxemia. LPS was responsible for the onset of metabolic diseases. In addition, changes of gut microbiota reduced metabolic endotoxemia and this effect correlated with decreased glucose intolerance, body weight gain, fat mass development, lower inflammation, and oxidative stress [26]. Our data demonstrate that consumption of high levels of dietary fat diet is accompanied by significant reduction in counts of lactic acid bacteria. As shown in Table 4, the administration of probiotic *Lactobacillus plantarum* LS/07 and *Lactobacillus plantarum *Biocenol LP96 caused an increase in the counts of LAB in caecum in comparison with the control group. Similarly, Xie et al. [27] demonstrated that a high fat diet changed the intestinal microflora composition; in particular, the number of *Lactobacillus* spp. was reduced. Cani et al. [26] showed that high fat diet reduced some Gram-positive and Gram-negative bacteria (*Lactobacillus *spp., *Bifidobacterium *spp., and *Bacteroides*-*Prevotella *spp.). Due to these changes, eating a high fat diet and lower counts of LAB influenced the intestinal microflora composition and metabolic processes in the caecum content, resulting in varied levels of *β*-glucuronidase in our experimental groups. Several studies have shown that probiotic bacteria reduce bacterial enzymes such as *β*-glucuronidase, *β*-glucosidase, azoreductase, nitroreductase, and other enzymes of intestinal microflora [15, 28]. The elevated activity of bacterial enzymes is associated with an increasing risk for various types of cancer. The results of the present study showed that enzymatic activity of *β*-glucuronidase was not significantly changed in experimental groups after application of probiotic microorganisms. Many studies have reported hypolipidemic and antiobesity effects of the same probiotic strains such as *Lactobacillus* spp. and *Bifidobacterium *spp. [29, 30]. We established a rat model based on a 10-week administration of high fat diet characterized by an increased body weight, ratio of the weight gain to the final weight, fat mass, and lipid parameters in serum and liver. Our results demonstrated that administration of the two *Lactobacillus* strains—*Lactobacillus plantarum *LS/07 and* Lactobacillus plantarum *Biocenol LP96—played a role in reducing serum TC, LDL-C, total bile acid, and non-HDL cholesterol. The possible mechanisms of probiotics involved in the hypolipidemic effect may be as follows: (1) the assimilation of cholesterol by bacterial growing cells; (2) the binding of cholesterol to the bacterial cellular surface, thereby inhibiting the absorption of cholesterol back into the body; (3) the deconjugation of bile acids by bacterial acid hydrolyses, increasing cholesterol excretion of deconjugated bile salts and increasing cholesterol uptake and metabolism in the liver as compensatory response because bile acids are synthesized from cholesterol in the liver; (4) inhibition of hepatic cholesterol and triglyceride synthesis through the action of short chain fatty acids, especially propionic acid [31–33].

The lipid levels in blood serum are regulated through absorption, synthesis, and excretion. High concentrations of total cholesterol and LDL cholesterol are highly associated with an increased risk of cardiovascular disease. Based on our results, different *Lactobacillus* strains lead to different responses of lipid parameters in serum. Compared with the HFD group, the LPH group had more decreased TC, LDL-CH, bile acids, and non-HDL levels such as LPP group. Non-HDL cholesterol is used as an estimation of the total number of atherogenic particles in plasma (VLDL + IDL (intermediate-density lipoprotein) + LDL) and relates well to apo B levels. Non-HDL cholesterol can provide a better risk estimation compared with LDL-CH, in particular in hypertriglyceridaemia combined with diabetes, the metabolic syndrome, or chronic kidney disease [34]. The oral administration of *Lactobacillus plantarum *LS/07 resulted in higher decreases of serum cholesterol and LDL cholesterol by 20% and 24%, whereas TG and VLDL levels were decreased by 39% in the LPP group. These results could be explained by confounding variable such as different sources and properties of lactobacilli strains. Harisa et al. [35] assume that hypotriglyceridemic effect of probiotics may be related to the initiation of lipases activity, decreasing intestinal absorption of lipids, or increasing lipid catabolism and/or antioxidants activity. Lipoprotein lipase is responsible for metabolism of TG consequently normalizing its plasma level. Moreover, reducing blood cholesterol and LDL cholesterol in hypercholesterolemic humans and animals lowers the incidence of cardiovascular disease. Therefore, lowering the LDL cholesterol level, such as main component of serum cholesterol, may be an important factor for reducing serum total cholesterol. LDL receptor controls blood cholesterol levels by hepatic absorption. Kumar et al. [36] demonstrated that LDL receptor mRNA expression was upregulated in the group supplemented with* Lactobacillus rhamnosus *GG. Similar results in rats had been reported by Park et al. [37] with *Lactobacillus acidophilus *ATCC 43121 supplementation. Synthesis of bile acids from cholesterol is the most important way of cholesterol excretion. They are synthesized in the liver in process that is regulated by many factors including nutrients, hormones, and bile acids [38]. The solubility of the hydrophobic steroid nucleus of primary bile acid (cholic and chenodeoxycholic acid) is increased by conjugation with glycine or taurine prior to secretion. Some probiotic strains have been found to produce bile salt hydrolase (BSH), enzyme that catalyses the hydrolysis of conjugated bile salts. Deconjugated bile salts are less reabsorbed from the intestinal lumen, which results in excretion of free bile acids in faeces. Deconjugation of bile acid by probiotic microorganism could lead towards a reduction in serum cholesterol by increasing new bile acids in liver or by reducing cholesterol solubility and thereby absorption of cholesterol from the gastrointestinal tract [39, 40]. The present study showed that oral administration of lactobacilli strains resulted in a reduction of serum total bile acids. *Lactobacillus plantarum* LS/07 significantly decreased (*P* < 0.05) total bile acids levels in serum. Similar results were also documented by Bertková et al. [41]. Usman and Hosono [42] studied supplementing the diet of hypercholesterolemic rats with different *Lactobacillus gasseri* strains and observed reduction in the serum bile acid concentration only in the animals that presented increased fecal excretion of these acids. Moreover in our study, the number of *Lactobacillus plantarum* LS/07 negatively correlated with total bile acids (Pearson's *r* = −0.54) and cholesterol (Pearson's *r* = −0.56), which suggested that these probiotic strain colonize efficiently rat gastrointestinal tract and thereby reduced serum cholesterol and total bile acid levels. Hepatic and fecal lipid contents were higher in high fat diet fed rats than in the experimental group (LPH and LPP). This suggests that the hypolipidemic effect of the *Lactobacillus* strains may be due to decreases in intestinal absorption of lipid or increases in lipid catabolism. Our lactobacilli strains did not appear to affect HDL cholesterol levels. These results are in accordance with those of various other workers [4, 27]. The finding that probiotic bacteria lower serum cholesterol and triglyceride concentrations is agreement with data from other studies [21, 43]. Several researchers St-Onge et al. [44] and Hatakka et al. [45] did not observe hypolipidemic effect from probiotic bacteria consumed by animals or human. These results may be due to the different properties of cultures used and other factors as bacterial ingestion dosage, cholesterol content in diet, animal used, and length of the feeding period [4].

In the present study, we observed that feeding of high fat diet nonsignificantly increases body weight and weight gain. The administration of *Lactobacillus plantarum* LS/07 and *Lactobacillus plantarum* Biocenol LP96 slightly increased body weight and weight gain. However, there was no significant difference in liver weight, but in LPH group there was significant lower ratio liver/body weight compared to HFD group. When comparing the body fat in HFD group to LPH and LPP groups, no significant difference was observed. Recent researches demonstrated that some probiotic bacteria have antiobesity effect and reduced body fat [28, 46]. Yin et al. [30] compared hypolipidemic and antiobesity effect of four bifidobacteria strains in obese rats. He observed that the four strains can reduce serum and liver triglyceride and total cholesterol, but *Bifidobacterium* M13-4 significantly improves body weight gain compared with control high fat diet group, while other *Bifidobacterium* strains more or less decrease body weight levels. In conclusion, he showed that *Bifidobacterium *M13-4 may generate a new conception: certain probiotics may promote body weight gain by more effective fat absorption, and a careful assessment is needed before probiotics therapy is given, especially in obese people.

## 5. Conclusion

Our *Lactobacillus *strains have variant hypolipidemic properties. In conclusion, these data show that administration of probiotics to high fat rats has hypolipidemic but no antiobesity effects: *Lactobacillus plantarum* LS/07 reduces serum cholesterol and LDL cholesterol levels, but *Lactobacillus plantarum* Biocenol LP96 decreases triglyceride levels and VLDL. Both probiotic strains reduce total bile acids in serum. These findings indicate that the effect of lactobacilli on lipid metabolism may differ among strains and that the *Lactobacillus plantarum* LS/07 and *Lactobacillus plantarum* Biocenol LP96 can be used to improve lipid profile and can contribute to a healthier bowel microbial balance.

## Figures and Tables

**Table 1:** Effect of lactobacilli strains on body weight, weight gain, and organ weight in rats.

	C	HFD	LPH	LPP
Initial BW (g)	365 ± 20.50^a^	354 ± 23.06^a^	360 ± 22.46^a^	350 ± 25.08^a^
Final BW (g)	440 ± 30.37^a^	461 ± 30.71^a^	483 ± 29.59^a^	488 ± 44.07^a^
Weight gain (g)	75 ± 10.57^b^	107 ± 11.84^ab^	123 ± 9.55^ab^	138 ± 20.54^a^
Ratio G/FBW (%)	16.27 ± 1.55^b^	22.82 ± 1.87^a^	25.4 ± 1.11^a^	26.94 ± 2.19^a^
Liver (g)	13.63 ± 0.94^a^	14.42 ± 1.20^a^	13.16 ± 0.93^a^	14.96 ± 1.41^a^
Ratio liver/FBW	3.12 ± 0.09^ab^	3.13 ± 0.17^a^	2.72 ± 0.07^b^	3.08 ± 0.06^ab^
Body fat (%BW)	16.27 ± 0.76^a^	21.62 ± 2.15^a^	20.72 ± 0.80^a^	22.26 ± 1.50^a^
Lean (%BW)	68.09 ± 0.91^a^	65.12 ± 5.58^ab^	63.34 ± 0.78^b^	62.41 ± 0.93^b^

Values represent mean ± SEM (*n* = 10).

^a,b,c^Mean values within a column with different superscript letters differ significantly (*P* < 0.05).

C: control group; HFD: high fat diet group; LPH: HFD + *Lactobacillus plantarum* LS/07; LPP: HFD + *Lactobacillus plantarum* Biocenol LP96.

BW: body weight; FBW: final body weight; G/FBW: gain/final body weight.

**Table 2:** Food intake, FER, and BWCH.

	C	HFD	LPH	LPP
Food intake (g)	23.65 ± 1.03^a^	18.91 ± 2.21^b^	17.46 ± 1.05^b^	18.93 ± 1.30^ab^
FER	3.05 ± 0.34^b^	5.69 ± 0.65^a^	7.06 ± 0.04^a^	7.00 ± 0.72^a^
BWCH (%)	19.78 ± 2.11^b^	30.28 ± 3.27^ab^	34.31 ± 1.99^a^	37.93 ± 3.91^a^

Values represent mean ± SEM (*n* = 10).

^a,b,c^Mean values within a column with different superscript letters differ significantly (*P* < 0.05).

C: control group; HFD: high fat diet group; LPH: HFD + *Lactobacillus plantarum* LS/07; LPP: HFD + *Lactobacillus plantarum* Biocenol LP96.

FER: feed efficiency ratio; BWCH: body weight change.

**Table 3:** Lipid parameters and non-HDL cholesterol.

	C	HFD	LPH	LPP
TC (mmol/L)	1.36 ± 0.07^b^	1.56 ± 0.05^a^	1.25 ± 0.05^b^	1.42 ± 0.04^ab^
TG (mmol/L)	0.72 ± 0.09^a^	0.67 ± 0.06^ab^	0.48 ± 0.04^bc^	0.41 ± 0.04^c^
LDL-CH (mmol/L)	0.22 ± 0.06^a^	0.33 ± 0.04^a^	0.25 ± 0.03^a^	0.34 ± 0.03^a^
VLDL (mmol/L)	0.33 ± 0.04^a^	0.30 ± 0.03^ab^	0.22 ± 0.02^bc^	0.18 ± 0.02^c^
HDL-CH (mmol/L)	0.82 ± 0.03^a^	0.92 ± 0.05^a^	0.78 ± 0.05^a^	0.89 ± 0.03^a^
Non-HDL-CH	0.54 ± 0.05^ab^	0.64 ± 0.04^a^	0.47 ± 0.03^b^	0.53 ± 0.03^ab^
Total bile acids (*µ*mol/L)	29.04 ± 5.47^a^	29.47 ± 5.14^a^	13.41 ± 1.48^b^	18.63 ± 2.08^ab^
Liver TCH (mg/g)	7.12 ± 0.61^a^	8.21 ± 1.01^a^	9.01 ± 0.99^a^	9.12 ± 1.02^a^
Liver TG (mg/g)	24.21 ± 2.41^b^	42.90 ± 3.11^a^	39.06 ± 2.36^a^	40.71 ± 3.01^a^
Fecal TCH (mg/g)	4.23 ± 0.51^b^	5.56 ± 0.98^ab^	6.98 ± 0.23^a^	6.01 ± 0.43^ab^
Fecal TG (mg/g)	5.11 ± 0.95^b^	12.26 ± 1.09^a^	13.78 ± 1.42^a^	13.91 ± 1.21^a^

Values represent mean ± SEM (*n* = 10).

^a,b,c^Mean values within a column with different superscript letters differ significantly (*P* < 0.05).

C: control group; HFD: high fat diet group; LPH: HFD + *Lactobacillus plantarum* LS/07; LPP: HFD + *Lactobacillus plantarum* Biocenol LP96.

TCH: total cholesterol; TG: triglyceride, LDL-CH: low density lipoprotein, VLDL: very low density lipoprotein; HDL-CH: high density lipoprotein.

**Table 4:** Population of lactic acid bacteria and activity of *β*-glucuronidase.

	C	HFD	LPH	LPP
Lactic acid bacteria	9.08 ± 0.051^a^	8.68 ± 0.214^b^	9.29 ± 0.052^a^	9.03 ± 0.122^ab^
*β*-Glucuronidase	0.077 ± 0.009^b^	0.110 ± 0.020^ab^	0.114 ±0.018^ab^	0.166 ± 0.015^a^

Values represent mean ± SEM (*n* = 10).

^a,b,c^Mean values within a column with different superscript letters differ significantly (*P* < 0 · 05).

C: control group; HFD: high fat diet group; LPH: HFD + *Lactobacillus plantarum* LS/07; LPP: HFD + *Lactobacillus plantarum* Biocenol LP96.

Lactic acid bacteria: log_10_ CFU/g; *β*-glucuronidase: *μ*mol/g/min.

## References

[1] Allender S, Scarborough P, Peto V (2008). *European Cardiovascular Disease Statistics*.

[2] McNamara DJ (2000). Dietary cholesterol and atherosclerosis. *Biochimica et Biophysica Acta*.

[3] Yang P-Y, Rui Y-C, Lu L (2005). Time courses of vascular endothelial growth factor and intercellular adhesion molecule-1 expressions in aortas of atherosclerotic rats. *Life Sciences*.

[4] Wang Y, Xu N, Xi A, Ahmed Z, Zhang B, Bai X (2009). Effects of *Lactobacillus plantarum* MA2 isolated from Tibet kefir on lipid metabolism and intestinal microflora of rats fed on high-cholesterol diet. *Applied Microbiology and Biotechnology*.

[5] Gao D, Zhu G, Gao Z, Liu Z, Wang L, Guo W (2011). Antioxidative and hypolipidemic effects of lactic acid bacteria from pickled chinese cabbage. *Journal of Medicinal Plant Research*.

[6] Homayouni A, Payahoo L, Azizi A (2012). Effects of probiotics on lipid profile: a review. *American Journal of Food Technology*.

[7] FAO/WHO (2002). *Guidelines for the Evaluation of Probiotics in Food*.

[8] Bomba A, Brandeburová A, Ričanyová J (2012). The role of probiotics and natural bioactive compounds in modulation of the common molecular pathways in pathogenesis of atherosclerosis and cancer. *Biologia*.

[9] Heyman M (2000). Effect of lactic acid bacteria on diarrheal diseases. *Journal of the American College of Nutrition*.

[10] Guarner F, Malagelada J-R (2003). Gut flora in health and disease. *The Lancet*.

[11] Saxami G, Ypsilantis P, Sidira M, Simopoulos C, Kourkoutas Y, Galanis A (2012). Distinct adhesion of probiotic strain *Lactobacillus casei* ATCC 393 to rat intestinal mucosa. *Anaerobe*.

[12] Shaper AG, Jones KW, Jones M, Kyobe J (1963). Serum lipids in three nomadic tribes of northern Kenya. *The American Journal of Clinical Nutrition*.

[13] Mann GV, Spoerry A (1974). Studies of a surfactant and cholesteremia in the Maasai. *American Journal of Clinical Nutrition*.

[14] Zavisic G, Petricevic S, Radulovic Z (2012). Probiotic features of two oral *Lactobacillus* isolates. *Brazilian Journal of Microbiology*.

[15] Strojný L, Bomba A, Hijová E (2011). Effects of a probiotic in combination with prebiotics on intestinal lactobacilli and coliforms and activities of bacterial enzymes in 1,2-dimethylhydrazine exposed rats. *Czech Journal of Animal Science*.

[16] Nemcová R, Bomba A, Gancarčíková S (2007). Effects of the administration of lactobacilli, Maltodextrins and fructooligosaccharides upon the adhesion of *E. coli* O8:K88 to the intestinal mucosa and organic acid levels in the gut contents of piglets. *Veterinary Research Communications*.

[17] Nemcová R, Borovská D, Koščová J (2012). The effect of supplementation of flax-seed oil on interaction of *Lactobacillus plantarum*—Biocenol LP96 and *Escherichia coli* O8:K88ab:H9 in the gut of germ-free piglets. *Research in Veterinary Science*.

[18] Juskiewicz J, Zdunczyk Z, Wroblewska M, Oszmianski J, Hernandez T (2002). The response of rats to feeding with diets containing grapefruit flavonoid extract. *Food Research International*.

[19] Friedewald WT, Levy RI, Fredrickson DS (1972). Estimation of the concentration of low-density lipoprotein cholesterol in plasma, without use of the preparative ultracentrifuge. *Clinical Chemistry*.

[20] Folch JM, Lees M, Solane SGH (1957). A simple method for the isolation and purification of total lipides from animal tissues. *The Journal of Biological Chemistry*.

[21] Wang C-Y, Wu S-C, Ng C-C, Shyu Y-T (2010). Effect of *Lactobacillus*-fermented adlay-based milk on lipid metabolism of hamsters fed cholesterol-enriched diet. *Food Research International*.

[22] Bomba A, Nemcová R, Gancarčíková S, Herich R, Guba P, Mudroňová D (2002). Improvement of the probiotic effect of micro-organisms by their combination with maltodextrins, fructo-oligosaccharides and polyunsaturated fatty acids. *British Journal of Nutrition*.

[23] Bäckhed F, Ding H, Wang T (2004). The gut microbiota as an environmental factor that regulates fat storage. *Proceedings of the National Academy of Sciences of the United States of America*.

[24] Turnbaugh PJ, Ley RE, Mahowald MA, Magrini V, Mardis ER, Gordon JI (2006). An obesity-associated gut microbiome with increased capacity for energy harvest. *Nature*.

[25] Cani PD, Amar J, Iglesias MA (2007). Metabolic endotoxemia initiates obesity and insulin resistance. *Diabetes*.

[26] Cani PD, Bibiloni R, Knauf C (2008). Changes in gut microbiota control metabolic endotoxemia-induced inflammation in high-fat diet-induced obesity and diabetes in mice. *Diabetes*.

[27] Xie N, Cui Y, Yin Y-N (2011). Effects of two *Lactobacillus* strains on lipid metabolism and intestinal microflora in rats fed a high-cholesterol diet. *BMC Complementary and Alternative Medicine*.

[28] An HM, Park SY, Lee DK (2011). Antiobesity and lipid-lowering effects of *Bifidobacterium* spp. in high fat diet-induced obese rats. *Lipids in Health and Disease*.

[29] Wang J, Zhang H, Chen X, Chen Y, Menghebilige M, Bao Q (2012). Selection of potential probiotic lactobacilli for cholesterol-lowering properties and their effect on cholesterol metabolism in rats fed a high-lipid diet. *Journal of Dairy Science*.

[30] Yin Y-N, Yu Q-F, Fu N, Liu X-W, Lu F-G (2010). Effects of four Bifidobacteria on obesity in high-fat diet induced rats. *World Journal of Gastroenterology*.

[31] Noh DO, Kim SH, Gilliland SE (1997). Incorporation of cholesterol into the cellular membrane of *Lactobacillus acidophilus* ATCC 43121. *Journal of Dairy Science*.

[32] Gill HS, Guarner F (2004). Probiotics and human health: a clinical perspective. *Postgraduate Medical Journal*.

[33] Liong MT, Shah NP (2006). Effects of a *Lactobacillus casei* synbiotic on serum lipoprotein, intestinal microflora, and organic acids in rats. *Journal of Dairy Science*.

[34] Reiner Ž, Catapano AL, De Backer G (2011). ESC/EAS guidelines for the management of dyslipidaemias. *European Heart Journal*.

[35] Harisa GI, Taha EI, Khalil AF, Salem MM (2009). Oral administration of lactobacillus acidophilus restores nitric oxide level in diabetic rats. *Australian Journal of Basic and Applied Sciences*.

[36] Kumar M, Rakesh S, Nagpal R (2013). Probiotic *Lactobacillus rhamnosus* GG and *Aloe vera* gel improve lipid profiles in hypercholesterolemic rats. *Nutrition*.

[37] Park YH, Kim JG, Shin YW, Kim SH, Whang KY (2007). Effect of dietary inclusion of *Lactobacillus acidophilus* ATCC 43121 on cholesterol metabolism in rats. *Journal of Microbiology and Biotechnology*.

[38] Fuchs M (2003). Bile acid regulation of hepatic physiology III. Regulation of bile acid synthesis: past progress and future challenges. *American Journal of Physiology*.

[39] Begley M, Hill C, Gahan CGM (2006). Bile salt hydrolase activity in probiotics. *Applied and Environmental Microbiology*.

[40] Lye H-S, Rahmat-Ali GR, Liong M-T (2010). Mechanisms of cholesterol removal by lactobacilli under conditions that mimic the human gastrointestinal tract. *International Dairy Journal*.

[41] Bertková I, Hijová E, Chmelárová A (2010). The effect of probiotic microorganisms and bioactive compounds on chemically induced carcinogenesis in rats. *Neoplasma*.

[42] Usman U, Hosono A (2000). Effect of administration of *Lactobacillus gasseri* on serum lipids and fecal steroids in hypercholesterolemic rats. *Journal of Dairy Science*.

[43] Minelli EB, Benini A, Marzotto M (2004). Assessment of novel probiotic *Lactobacillus casei* strains for the production of functional dairy foods. *International Dairy Journal*.

[44] St-Onge M-P, Farnworth ER, Savard T, Chabot D, Mafu A, Jones PJH (2002). Kefir consumption does not alter plasma lipid levels or cholesterol fractional synthesis rates relative to milk in hyperlipidemic men: a randomized controlled trial. *BMC Complementary and Alternative Medicine*.

[45] Hatakka K, Mutanen M, Holma R, Saxelin M, Korpela R (2008). *Lactobacillus rhamnosus* LC705 together with *Propionibacterium freudenreichii* ssp shermanii JS administered in capsules is ineffective in lowering serum lipids. *Journal of the American College of Nutrition*.

[46] Sato M, Uzu K, Yoshida T (2008). Effects of milk fermented by *Lactobacillus gasseri* SBT2055 on adipocyte size in rats. *British Journal of Nutrition*.

